# Targeting Innate-Like T Cells in Tuberculosis

**DOI:** 10.3389/fimmu.2016.00594

**Published:** 2016-12-21

**Authors:** Shouxiong Huang

**Affiliations:** ^1^Department of Environmental Health, University of Cincinnati College of Medicine, Cincinnati, OH, USA

**Keywords:** vaccine, innate-like T cells, *Mycobacterium tuberculosis*, lipid, riboflavin metabolites, CD1, MR1, antigen presentation

## Abstract

Peptide-specific conventional T cells have been major targets for designing most antimycobacterial vaccines. Immune responses mediated by conventional T cells exhibit a delayed onset upon primary infection and are highly variable in different human populations. In contrast, innate-like T cells quickly respond to pathogens and display effector functions without undergoing extensive clonal expansion. Specifically, the activation of innate-like T cells depends on the promiscuous interaction of highly conserved antigen-presenting molecules, non-peptidic antigens, and likely semi-invariant T cell receptors. In antimicrobial immune responses, mucosal-associated invariant T cells are activated by riboflavin precursor metabolites presented by major histocompatibility complex-related protein I, while lipid-specific T cells including natural killer T cells are activated by lipid metabolites presented by CD1 proteins. Multiple innate-like T cell subsets have been shown to be protective or responsive in mycobacterial infections. Through rapid cytokine secretion, innate-like T cells function in early defense and memory response, offering novel advantages over conventional T cells in the design of anti-tuberculosis strategies.

Host immune responses are critical in controlling pandemic and life-threatening *Mycobacterium tuberculosis* (*M. tuberculosis*) infection in humans through the development of protective immunity in a T cell-dependent manner (1–3). An effective T cell response leads to a low lifetime risk of developing active tubercular diseases. In mice, the activation of CD4^+^ or CD8^+^ T cells is important for maintaining a low bacterial load in tissues, as shown using antibody depletion and T cell adoptive transfer in various T cell-deficient mice (4–9). In humans, the importance of CD4^+^ T cell response is supported by the dramatically increased risk of active tuberculosis with the co-infection of human immunodeficiency virus (HIV), which reduces the number of CD4^+^ T cells in patients (10). Abundant circumstantial evidence also supports that the effector functions of human CD8^+^ T cells are able to suppress mycobacterial growth (3), although the activating elements for different CD8^+^ T cell populations remain elusive (3, 11, 12). Thus, therapeutic and vaccine strategies aimed at the development of protective T cell responses would be beneficial for minimizing the treatment course of antibiotics, preventing the spread of drug-resistant *M. tuberculosis*, and reducing lung inflammatory responses and injury (13).

The licensed vaccine Bacillus Calmette–Guérin (BCG) has saved many children’s lives, despite offering insufficient protection against pulmonary tuberculosis in adults and no evidenced efficacy in controlling the prevalence of tuberculosis (14). Upon discovery of virulence-associated genetic complexes from *M. tuberculosis*, virulent factors encoded by these pathogenicity islands have been shown to induce protective T cell responses (15, 16). Many vaccination approaches in the pipeline of development are based on immune response mediated by these virulent proteins through antigen presentation by major histocompatibility complex (MHC) class I or MHC class II molecules (15, 17, 18). Immune responses mediated by conventional T cells feature highly specific and heterogeneous immune responses that are determined by tri-molecular interactions among highly polymorphic antigen-presenting molecules, diversified antigenic peptides, and variable T cell receptors in human populations (19). Meanwhile, conventional T cells require a relatively long priming stage allowing naïve T cells to differentiate into effector cells with antimycobacterial functions. As discussed below, innate-like T cells display conserved receptor interaction and fast-responding kinetics in antigen presentation and effector responses, which contribute to unique antimycobacterial immune defenses.

## Discoveries of Unconventional T Cells in *M. Tuberculosis* Infections

Until recently, CD8^+^ mucosal-associated invariant T (MAIT) cells in antimycobacterial responses were difficult to distinguish from conventional CD8^+^ T cells. High-frequency CD8^+^ T cells in both infected and uninfected individuals are reactive to *M. tuberculosis*, potentially challenging the notion that CD8^+^ T cells are conventional T cells activated by mycobacterial peptides in infected humans. Limited dilution analysis showed that a high percentage of these *M. tuberculosis*-reactive CD8^+^ T cells were not restricted by classical MHC class I molecules (12, 20). Surprisingly, these non-conventional T cells, which covered 80% of the total CD8^+^ T cell clones generated from individual healthy donors, 70% from latently infected donors, and about 35% from donors with active tuberculosis, were identified as MAIT cells in a blocking assay using an antibody against the MHC-related protein I (MR1) (12). This finding indicates that a high percentage of CD8^+^ T cells are not restricted by MHC class I proteins and do not respond to peptide antigens. In fact, they are restricted by MHC class I-like MR1 protein, as confirmed with functional blockage using an anti-MR1 antibody (12, 20). The existence of a high percentage of mycobacterial-reactive CD8^+^ T cells in uninfected healthy humans is also striking, as the stimulants for these CD8^+^ T cells must be shared between non-mycobacterial and mycobacterial organisms, and peptide antigens are least likely to explain this result. To date, we know MAIT cells respond to riboflavin precursor metabolites produced by a variety of bacterial species, including *M. tuberculosis* (21). Concurrently, MAIT cells have been shown to be protective against mycobacterial infection using infected mouse models deficient of MR1 protein or with overexpression of the MAIT cell TCR (20).

The presence of antimycobacterial T cells restricted by the cluster of differentiation I (CD1) proteins has been reported along with the initial discovery of a CD1 antigen presentation function (22–26). The expression of an invariant TCR sequence likely supports a unique activation mechanism diverted from conventional T cells (27). Indeed, CD1-restricted T cells from peripheral blood can be stimulated by autologous immature CD1^+^ dendritic cells and respond at a significant magnitude and frequency in asymptomatic *M. tuberculosis*-infected donors (23). It appears that lipid-stimulated T cell proliferation is minimally detectable or absent in the blood samples from active tuberculosis patients and become detectable 2 weeks after the start of antibiotic treatment. This interesting finding suggests that *M. tuberculosis*-reactive CD4^+^ T cells respond to *M. tuberculosis* lipid antigens presented by CD1 proteins and exist abundantly in healthy individuals with previous exposure to *M. tuberculosis* (23).

## A Glance at Unconventional T Cells

Unlike conventional T cells, which are restricted by the antigen-presenting molecules encoded by the MHC genetic complexes, unconventional T cells are activated by MHC class I-like molecules that are encoded by genes outside the MHC complexes. As shown in Table 1, unconventional T cells are mostly restricted by CD1 and MR1 proteins. Specifically for two major invariant T cell populations, MAIT cells are activated by riboflavin precursor metabolites presented by the MR1 protein, and natural killer T (NKT) cells are activated by various lipid metabolites presented by the CD1d protein (Table 1). CD1- and MR1-restricted T cell subsets are in fact abundant in human peripheral blood or tissues. In particular, MR1-restricted MAIT cells and CD1a- and CD1c-restricted T cells are highly frequent in human blood (12, 28, 59); MAIT cells and iNKT cells are also abundant in human liver tissues (29, 30). The functional uniqueness of MAIT and iNKT cells is mostly attributable to their invariant TCRα sequences, which were initially characterized in the early 1990s (27). The expression of invariant TCRα chains with biased usage of TCRβ chains is now known as a major feature in MAIT, iNKT, and other unconventional T cell populations (Table 1), contributing to the quick-responding kinetics described below.

**Table 1:** **Antigen-presenting molecules, antigens, and TCRs for unconventional T cells**.

Restriction	Named subsets	TCRα	TCRβ	Mammalian antigens	Microbial antigens
*hMR1/mMR1*	Mucosal-associated invariant T	Invariant TRAV1-2	Biased TRBV6-1, TRBV20-1	Unknown	Riboflavin precursor metabolites (31–37)
*hCD1d/mCD1d*	iNKT	Invariant TRAV10 and TRAV27	Biased TRBV25-1	iGb3 (38), ganglioside (39), ether-lysophosphatidic acid, plasmalogen lysophosphatidylethanolamine (40), lysophosphatidylcholine (41), phosphatidylinositol, phosphatidylglycerol, phosphatidylethanolamine (42), α-galactosylceramides (43)	α-Galactosylceramides (*Agelas*) (44), (*Bacteroides*) (45), Asperamide B (*Aspergillus*) (46), cholesteryl α-glycoside (*Helicobacter*) (47), α-galactosyldiacylglycerol (*Borrelia*) (48), α-glucosyldiacylglycerol (*Streptococcus*) (49), α-galacturonosylceramide, α-glucuronosylceramide (*Sphingomonas*) (50–52)
*hCD1d/mCD1d*	dNKT	Diverse or oligoclonal TRAV17, TRAV13 TRAV7, TRAV9	Biased TRBV12	Lysosphingomyelin, lysophosphatidyethanolamine, phosphatidylglycerol, phosphatidylinositol, phosphatidylethanolamine β-glucosphingomyelin, cardiolipin (42, 53), sulfatides (54), ganglioside (55)	phosphatidylglycerol, cardiolipin, phosphatidylinositol (56, 57)
*hCD1a*		Diverse	Diverse	lysophosphatidylcholine (58) sulfatides (54) squalene, wax esters, triacylglycerides (59)	Dideoxymycobactin^a^
*hCD1b*	GEM	Invariant TRAV1-2	Biased TRBV6-2		Glucose monomycolate^a^ (25, 60)
	LDN5-like T	Biased TRAV17	Biased TRBV4-1		Glucose monomycolate^a^ (25, 61)
		Diverse	Diverse	Sulfatides (54) ganglioside (39) phosphotidylglycerol (61)	Sulfoglycolipids^a^ (62), diacylated sulfoglycolipids^a^ (63), glycerol monomycolate^a^ (64), phosphotidylglycerol (61), lipoarabinomannan^a^, phosphatidylinositol mannosides^a^ (65)
*hCD1c*		Diverse	Biased TRBV7-8, TRBV7-9	Sulfatides (54), methyl-lysophosphatidic acid (66)	Mannosyl-phosphomycoketide^a^ phosphomycoketide^a^ (67)
*HLA-E/Qa-1*		Biased	Biased	Major histocompatibility complex class I leader peptide, HSP60 peptide	GroEL mycobacterial^a^ peptides (68)
*HLA-A, B, C/H-2K, D, L*		Diverse	Diverse	Various self peptides	ESAT-6^a^, Ag85B^a^, TB10.4^a^ peptides (15, 16)

*HLA, human leukocyte antigen; Qa-1, a nonclassical MHC class I protein in mice; H-2, the MHC complex in mice; dnkt, diverse natural killer T cells; GEM, germline-encoded, mycolyl lipid-reactive T cells; LDN5, name of a T cell line; TRAV, T cell receptor gene α chain variable region; TRBV, T cell receptor gene β chain variable region; iGb3, isoglobotrihexosylceramide*.

*Mycobacterial antigens are labeled with ^a^ and identified antigens are referenced*.

The identification of mycobacterial antigens for unconventional T cell activation has focused on the lipid antigens presented by group I CD1 proteins (CD1a, CD1b, and CD1c). Unconventional T cells against *M. tuberculosis* were in fact initially discovered to respond to CD1b-restricted mycobacterial lipid antigen (22). Thereafter, more antimycobacterial lipid-specific T cells were discovered to detect mycobacterial lipid antigens presented by group I CD1 proteins (Table 1). Subsets of CD1a-restricted T cells, represented by the cell line CD8-2, are reactive to dideoxymycobactin (DDM) (24). CD1b-restricted T cells are able to recognize more complex mycobacterial lipids, including glycerol monomycolate (64), glucose monomycolate (25), free mycolic acid (69), diacylated sulfoglycolipids (63), and phosphatidylinositol mannosides (70). Several lines of CD1c-restricted T cells have also been derived in response to a different class of mycobacterial lipid, mycoketides, including the T cell lines CD8-1, which responds to mycobacterial β-mannosyl phosphomycoketide from mycobacterial lipid extracts, and DN-6, which recognizes phosphomycoketide (26, 67). As summarized, these unconventional T cells exhibit different features from conventional T cells in antigen presentation (Table 1).

## Innate-Like Postulate and Fast-Responding Kinetics

**Figure 1:**
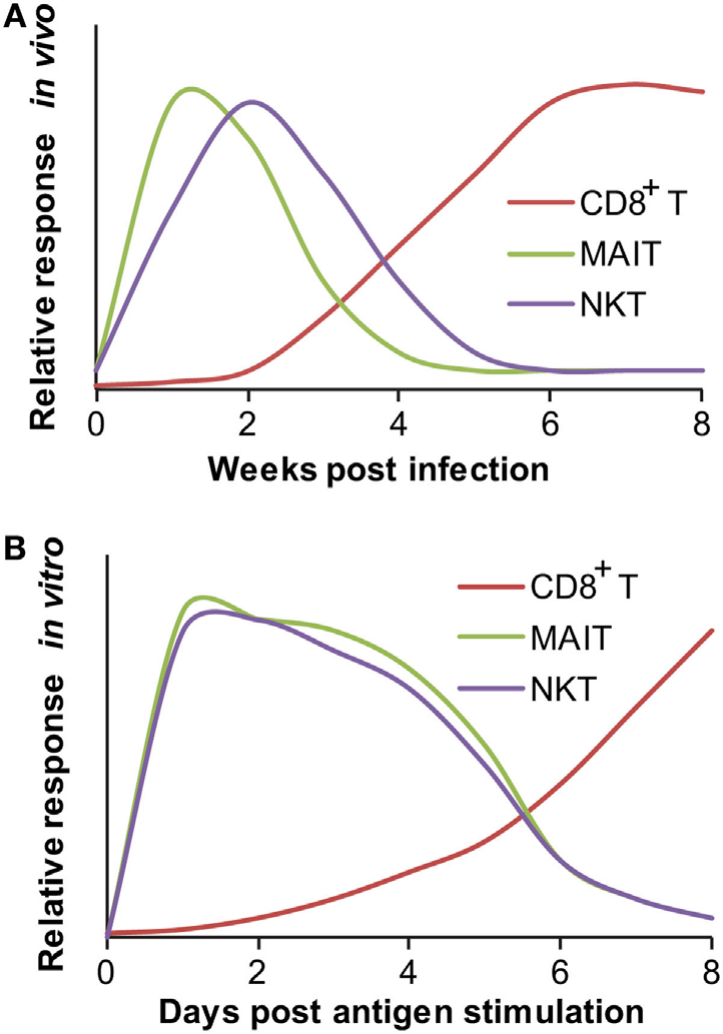
**Responding kinetics of mucosal-associated invariant T (MAIT) and natural killer T (NKT) cells**. **(A)** *In vivo* responding kinetics were hypothesized based on the presence of mycobacterial antigen-specific CD8^+^ T cells in lung tissues (3), the ability of MAIT cells to inhibit Bacillus Calmette–Guérin (BCG) growth in lung tissues (80), and the ability of transferred iNKT to inhibit *M. tuberculosis* growth in lung tissues (81). **(B)** *In vitro*-responding kinetics were estimated according to the acquisition of cytolytic function by CD8^+^ T cells upon *in vitro* peptide stimulation (77), cytokine production by polyclonal MAIT cells upon stimulation with BCG-infected macrophages (80), and cytokine production by tetramer-isolated human polyclonal NKT cells upon antigen-specific activation (82).

To consider T cell populations as being innate-like requires comparison of the biological features of T cells with those of cells from the innate and adaptive immune systems (Table 2). One measurable characteristic of an innate-like postulate is the quick activation kinetics from pathogen-unexposed precursors or naïve cells to effector cells in an antigen-specific manner. The activation of conventional naïve T cells requires prolonged antigenic priming for days and weeks following a primary infection to stimulate clonal expansion and effector function (71). Conventional CD8^+^ T cells have been shown with antimycobacterial responses, as supported by the *M. tuberculosis*-infected β2m^−/−^ and TAP1^−/−^ mice, which are unable to control *M. tuberculosis* replication in the lung and cause premature death (2, 72, 73). These mycobacterial peptide-specific CD8^+^ T cells occur in the draining lymph node, become detectable in lung tissues within 2 weeks, and peak around 5–8 weeks in lung tissues after a primary infection in mice (Figure 1A) (3). Using a skin test, immune responses can be detected 5–6 weeks after *M. tuberculosis* infection in humans (74). In a drastic contrast to fast T cell responses to other intracellular bacteria, such as *Listeria* infection in mouse spleen (75), a much slower kinetics of T cell responses in *M. tuberculosis* infection may attribute to a slower growing curve of *M. tuberculosis*, bacterial inhibition of migratory activity, bacterial modulation of host antigen-presenting cells, and relatively immunoprivileged nature of the healthy alveolar tissues (2, 76). The antigen stimulation for conventional T cell activation occurs faster *in vitro* than *in vivo* because sufficient antigen presentation is usually warranted when setting up cell culture. In cell culture, mouse naïve CD8^+^ T cells require antigen stimulation for at least a week *in vitro* to observe the cytotoxic activity to specific antigens (77). In fact, several rounds of stimulation with peptide-pulsed macrophages for multiple weeks are required to achieve stronger responses of naïve CD8^+^ T cells, such as the generation of anti-HIV cytolytic T cells (78). Distinctively, the response of macrophages and other innate immune cells to uptake bacterial materials for priming adaptive immune responses is believed in a very early stage, generally within hours, after infection (79).

**Table 2:** **Characteristics of innate-like T cells**.

	Innate immune cells	Innate-like T cells	Conventional T cells
Cell examples	Macrophages, dendritic cells, natural killer cells, granulocytes	Mucosal-associated invariant T (MAIT) cells, invariant nature killer T (iNKT) cells, γδT cells, CD1-restricted T subsets, HLA-E-restricted T cells	Conventional αβT cells, B cells
Activation elements	PRR (e.g., TLR, NLR)	Major histocompatibility complex (MHC) class I-like molecules	MHC molecules
Pre-activated	Yes	Yes	No
Antigen-presenting molecules	No	Highly conserved	Highly polymorphic
Antigen specificity	No	Low specificity	High specificity
Activation kinetic *in vivo*	Quick (hours)	Quick (hours to days)	Slower (days to weeks)
Receptors	Highly conserved	Highly conserved or less variable	Highly variable
Precursor frequency	High	High	Low
Diversity in responses	Low	Low in the same subset	High
Memory	No	Pre-formed	Yes

*PRR, pattern recognition receptor; TLR, toll-like receptors; NLR, nucleotide-binding oligomerization domain-like receptors (NOD-like receptor); HLA, human leukocyte antigen*.

Unlike conventional T cells, innate-like T cells, such as MAIT cells, are ready to respond to mycobacterial or other antigens within hours to days (83–85). When pathogen-unexposed mouse MAIT cells were cocultured with BCG-infected macrophages, the production of a large amount of IFN-γ and IL-17a was detectable from MAIT cells within 4 days of coculture (80), although the time course needs to be further determined and the actual responding time is likely shorter. The early *in vitro* response has also been supported by the upregulation of CD69 expression on human and mouse MAIT cells following overnight incubation with *E. coli-* or *Mycobacterium abscessus*-infected monocytes (12, 20), or *M. tuberculosis-*infected lung epithelial cells (12). The secretion of IFN-γ (12, 20) and tumor necrosis factor-α (TNF-α) (12, 86) upon MAIT cell activation can be observed as well after incubating bacterial-infected antigen-presenting cells and pathogen-unexposed MAIT cells for 16–48 h (Figure 1B). This *in vitro* responding kinetics of MAIT cells is much faster than that of naïve conventional T cells stimulated with peptide antigens, which usually need 7 days or more to exhibit proliferation or effector function (78, 87). *In vivo*, MAIT cell accumulation in the primary infected lung tissue starts less than 10 days post BCG infection, as detected by an MR1-tetramer for measuring the frequency of MAIT cells isolated from bronchoalveolar lavage fluid (BALF) and lung tissue (33). Taking into account the slow growing curve and immune-escaping mechanism mediated by *M. tuberculosis* as discussed (2, 76), the kinetics of MAIT cell responses is essentially faster than the response of conventional CD8^+^ T cells in the lung infection of *M. tuberculosis* (Figure 1A). It appears that both the *in vitro* and *in vivo* kinetics of MAIT cells upon mycobacterial infection can be considered to resemble that of conventional memory T cells (33, 88).

In addition to MAIT cells, other unconventional T cells also exhibit quick-responding kinetics in their “primary” responses, including various CD1-restricted T cell subsets and HLA-E-restricted T cells (Table 1). NKT cells were identified in response to mycobacterial infection by the fact that the adoptive transfer of naïve splenic iNKT cells from uninfected mice resulted in a significantly reduced burden of *M. tuberculosis* in the lungs of infected mice (81). This adoptive transfer assay provided evidence that CD1d-restricted NKT cells mediate protection against *M. tuberculosis in vivo* using innate-like responding kinetics, which allows visualization of the inhibition curve of *M. tuberculosis* within 3 weeks after the transfer of naïve iNKT cells, supporting the quick-responding kinetics of naïve iNKT cells (81). As characterized, the iNKT antigen α-galactosylceramide (α-GalCer) also ameliorated the course of tuberculosis in mice in the early stage of *M. tuberculosis* infection but provided no additional survival benefit for an extended application (89). Although the lack of long-term protection of α-GalCer in the mycobacterial-infected mouse model is difficult to explain, it is perhaps consistent with the observation that CD1d^−/−^ mice are not more sensitive to *M. tuberculosis* infection (8). Moreover, the protection provided by iNKT cells appears similar to the protection offered by MAIT cells for decreasing BCG growth in lung tissues at 10 days of infection rather than at 30 days. These initial results support that innate-like MAIT and iNKT cells provide protection against mycobacterial infection in the early stage of infection. Although MAIT cells produce more cytokines at 30 days, whether innate-like T cells are able to further control the bacterial growth in later stages, cross-talk with innate immune cells, bridge the activation of conventional T cells, and regulate inflammatory responses in infected tissues are interesting topics that require further investigations (80).

The kinetics for group I CD1-restricted T cells in responding to mycobacterial and other antigens remain poorly understood, mainly due to the lack of expression of human CD1a, CD1b, and CD1c homologs in mouse models. Interestingly, a recent study using humanized mice showed that the kinetics of primary activation and memory response of group I CD1-restricted T cells were somewhat delayed (90). The antimycobacterial immune responses of group I CD1-restricted T cells peaked at 7 days following the immunization of *M. tuberculosis* lipids and showed a bit more rapid secondary responses at 5 days (90). Whether naïve group I CD1-restricted T cells are able to quickly respond to mycobacterial infections similarly to iNKT cells and have an innate-like response *in vitro* or *in vivo* remains unknown.

The innate-like kinetics of activation for unconventional T cells are correlated to highly conserved structures and interacting modes of three types of key molecules, antigen-presenting molecules, antigens, and TCRs, which are variable for conventional T cell activation. As detailed below, the conserved genetic feature of each type of molecules is not restricted by the individual donor and will contribute to promiscuous tri-molecular interaction in a donor-unrestricted manner, providing an immunogenetic basis for explaining innate-like responses in unconventional T cells (91).

## Highly Conserved Antigen-Presenting Molecules

In 1989, Janeway hypothesized that the innate immune system is able to regulate adaptive immune responses through innate immune recognition, such as pattern recognition (92). This prediction has been validated and has contributed to the understanding of the regulatory role of the innate immune system in adaptive immune responses (93). The innate immune system displays a remarkable homology of various innate immune molecules, including pattern recognition receptors (PRR) such as toll-like receptors (TLRs), which either recognize pathogen-associated microbial patterns (PAMP) or endogenous damage-associated molecular patterns (DAMP) (94–96). The common feature of these innate immune receptors is that they are germline-encoded and are considered highly conserved, with limited single-nucleotide polymorphisms (SNPs) in humans (97, 98). Innate cells with PRR expression are capable of mounting rapid effector responses independently of clonal expansion. This strategy of early pathogen detection in the innate immune system contrasts with that in the adaptive immune system. The HLA gene complex is highly diverse, with more than 3,000 variant alleles discovered in the HLA-A, B, or C loci, to date, in human populations, and it can also form 15,000–70,000 possible α and β chain combinations for HLA-DQ, DR, and DP molecules (from the IMGT/HLA website: http://www.ebi.ac.uk/ipd/imgt/hla/stats.html, September 13, 2016). This profound heterogeneity confers hypervariable interaction in antigen presentation for conventional T cell activation in human populations and is also functionally reflected in the difficulty of finding non-rejected graft donors for organ transplantation except for identical twins (99).

Similar to PRR molecules and drastically different from conventional HLA proteins, HLA class I-like proteins, such as MR1, CD1, and HLA-E, are considered to be highly conserved in humans (85, 91, 100, 101). Few SNPs have been identified by gene sequencing for HLA class I-like proteins, although more will likely be identified when human genome sequences are further available. The *MR1* gene is generally considered invariant in humans, except for two silent mutations, one of which generates a STOP codon in the α2 domain for an *MR1* pseudogene (102). Different splicing variants of the *MR1* gene have also been identified in other mammals (100, 103). The sequences of the ligand-binding and TCR-interacting domains (α1 and α2 domains) of the MR1 protein are identical between humans and chimpanzees, and they are highly homologous in mammals (100). It is difficult to imagine that human populations have such sequence variation, but humans and chimpanzees share the same sequence in the α1 and α2 domains of the MR1 protein. In regard to *CD1* genes, SNPs in the α1 domain (104, 105) and the non-coding regions (106) of the human *CD1a* and *CD1d* genes have been identified, and non-synonymous mutations have also been deduced from exon 2 of both genes (107). Few identified SNPs support that *MR1* and *CD1* genes are still very conserved and appear similar to innate immune receptors such as TLRs in terms of sequence stability in human populations. Although these rare *MR1* or *CD1* variants may exist in some individuals, nearly monomorphic sequence of MHC class I-like molecules in humans provides an almost identical platform for antigen presentation and T cell activation in *M. tuberculosis* infections. Thus, a vaccination strategy designed based on these highly conserved structures of antigen-presenting molecules would be highly applicable to a broad range of the human population, in contrast to vaccination strategies based on the classical HLA system (108, 109).

## Cross-Species Conservation of Non-Peptidic Antigens

Since the discovery of T cells using neonatally thymectomized mice in 1961 (110, 111), synthetic polypeptides have been used as model compounds for the elucidation of the molecular basis of immune responses (112), and peptide antigens have been shown to interact with MHC proteins for T cell activation (113). Peptides are probably better signatures for distinguishing non-self from self and fit nicely to the immunological paradigm in which high specificity for recognition and clearance of non-self is a major theme. In contrast to the chemical specificity of peptide compounds, lipids, and other small metabolites with the same or similar structures at least partially shared by humans, mammals, and even microbes are perhaps less consistent with the immunological theory of “self–non-self discrimination.” Interestingly, these highly conserved and structurally similar metabolites cross organisms, similar to PAMP or DAMP, which bind to innate receptors, are products of the fundamental metabolic pathways that are critical for cellular structures and functions in organisms. For the development and expansion of MAIT cells, the molecular factors provided by commensal bacteria are critical because MAIT cells are less detectable in germ-free mice (114). The result that germ-free mice do not demonstrate expanded MAIT cells suggests that microbial factors are critical for the activation and development of MAIT cells. Recently, bacterial metabolites in vitamin B2 and B9 metabolism were identified as MAIT cell antigens or ligands associated with the MR1 protein (31, 34). The first category of MR1 ligands, 6-formylpterin (6-FP) and acetyl-6-formylpterin (Ac-6-FP) as intermediate metabolites from vitamin B9 (folic acid) metabolism, has been identified from culture media. These two ligands function as antagonist molecules for blocking known MAIT cell activation (31, 35). The second category of MR1 ligands derives from the precursor metabolites in vitamin B2 (riboflavin) metabolism and includes three ribityllumazine species, 7-hydroxy-6-methyl-8-d-ribityllumazine (RL-6-Me-7-OH), 6,7-dimethyl-8-d-ribityllumazine (RL-6,7-diMe), and reduced 6-hydroxymethyl-8-d-ribityllumazine (rRL-6-CH2OH). These ligands exist in bacterial culture supernatant (31, 115) and have a bicyclic core similar to the formyl-pterins but are functional as agonists for stimulating MAIT cells when presented by the MR1 protein. The presence of a ribityl side chain from ribityllumazine species is critical for interacting with TCR for MAIT cell activation (31, 34, 35). A recently added third category of MR1 ligands includes two pyrimidine metabolites in riboflavin metabolism, 5-(2-oxopropylideneamino)-6-d-ribitylaminouracil (5-OP-RU) and 5-(2-oxoethylideneamino)-6-d-ribitylaminouracil (5-OE-RU) (36), which are considered to be the most potent known MAIT cell activators. Interesting research from Australia and France used bacterial strains defective in enzymes for riboflavin synthesis to show that mutations in the upstream genes involved in the synthesis of 5-amino-6-d-ribitylaminouracil (5-A-RU), the precursor of 5-OP-RU and 5-OE-RU, ablate MAIT cell activation by bacterial supernatants, whereas mutated genes involved in reactions downstream of 5-A-RU do not (36, 116, 117).

These precursor metabolites in riboflavin metabolism for MAIT cell activation provide several unique features that are different from those of peptide antigens for conventional T cells. First, the same or a similar group of riboflavin precursor metabolites is produced in a wide variety of bacteria and fungi, such as *Escherichia coli, Pseudomonas aeruginosa, Klebsiella pneumoniae, Staphylococcus aureus, Salmonella enterica* serovar Typhimurium, *M. tuberculosis*, and *Candida albicans*, but not in *Listeria monocytogenes* and certain strains of *Enterobacter* or *Streptococcus* (20, 31, 32). It appears that riboflavin biosynthesis is a canonical pathway for these stimulatory bacterial species and involves a number of enzymes essential for producing MAIT cell antigens (37). Second, the riboflavin pathway is essential for the survival and biological function of many bacteria (118–120). These metabolite antigens, which are generated by commensal bacteria or pathogenic microorganisms, are presumably pre-existing in humans unexposed to other pathogens, such as *M. tuberculosis*. If this hypothesis is true, MAIT cells likely have encountered the shared bacterial riboflavin metabolites in individuals uninfected by *M. tuberculosis*. Thus, these metabolite antigens derived from non-mycobacterial species contribute to the pre-activated state of precursor MAIT cells. Upon the primary infection of *M. tuberculosis*, these pre-activated host MAIT cells are able to quickly respond to the same or similar antigens derived from *M. tuberculosis*. Third, the presentation of small metabolites does not require the processing of proteasome or endocytic peptide digestion which is needed for peptide antigen presentation mediated by MHC class I and class II molecules (121). However, the production of bacterial and host small molecules connects antigen presentation to the metabolism of various lipid and vitamin metabolites, which are involved in broad biological functions. Finally, metabolite antigens will result in a chemically conserved interaction with antigen-presenting molecules and TCRs, in which TCR contact will be less dependent on antigen structures, as described below.

Unlike conventional peptide antigens, lipid antigens for CD1-restricted T cells also show some cross-species similarities that contribute to the innate-like responses of CD1-restricted T cells. Recently, lipodomic analyses have identified a broad range of lipid metabolites that associate with CD1a, CD1b, CD1c, and CD1d proteins. These lipid metabolites broadly include various species of sphingolipids, glycosphingolipids, glycerophospholipids, lysophospohlipids, ether-linked phospholipids, acylglycerols, wax esters, and fatty acids, as reported and reviewed (41, 59, 122–125). Similar interesting features are usually shared by lipid metabolites that are identified from different bacteria or between mammals and bacteria (Table 1). For example, NKT cells respond to an exogenous α-galactosylceramide that was originally identified in the marine sponge *Agelas mauritianus* (44). Although most glycosyl sphingosines in mammals show a β-anomeric linkage, α-glycosylceramides were also recently detected in mammalian cells (43). Similar lipid antigens, glycosphingolipids, have been identified in various bacterial species, including non-pathogenic *Sphingomonas* (19, 50, 51) and *Bacteroides* species that are part of the gut microbiota (45, 126–128). Although bacterial and mammalian lipid antigens presented by CD1 proteins display some structural similarities, *Mycobacterium* spp. are considered highly unique in lipid metabolites. Several identified CD1-presented lipid antigens, including CD1a-presented mycobactin metabolites (24), CD1b-presented monomycolate, mycolic acid, sulfoglycolipids (25, 63, 64, 69), and CD1c-presented phosphatidylinositol mannosides (70), and mannosyl phosphomycoketide (26), have not yet been fully investigated in terms of their cross-reactivity to a high percentage of lipid-specific T cells.

To bind to MHC class I proteins, nonamer peptides optimally fit to the length of the groove in MHC class I proteins and longer peptides (~14–20mers) extend beyond the whole length of the ligand-binding groove in MHC class II proteins (109, 121, 129, 130). Dependent on the ionic and hydrogen bonding interactions between peptides and MHC proteins, the anchor residues from antigenic peptides and the corresponding binding pockets of MHC proteins can be defined (131–133). As a result, classical MHC proteins discriminate specific residues of antigenic peptides and their positions in the peptide sequence. This recognition is determined by the products of genetic codes, which are subject to mutation, genetic inheritance, and functional selection. However, antigen binding to an MHC class I-like protein uses a manner of interaction different from that of MHC–peptide interaction.

## A Cyclic Anchor and Central Portal for MR1

**Figure 2:**
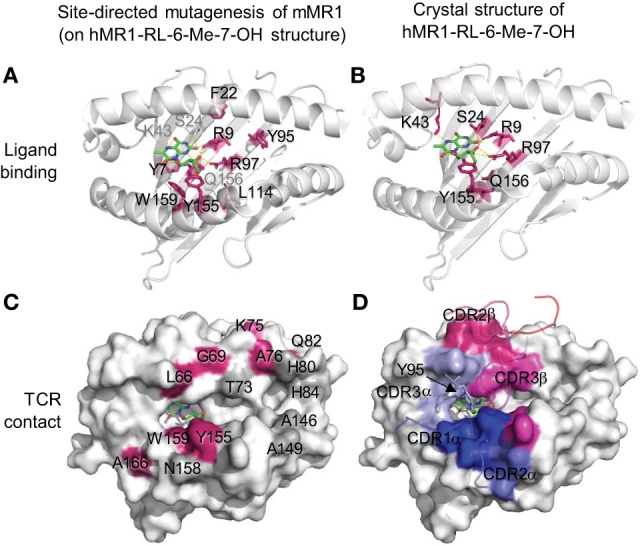
**Ligand-binding and TCR contact of MR1 protein inferred from mutagenesis (84) and structural (34) studies**. **(A)** Mutated mouse MR1 residues (84) are annotated on a human MR1 crystal structure (hMR1-RL-6-Me-7-OH) (34) to show the resulted functional impact on MR1 surface expression and mucosal-associated invariant T (MAIT) cell activation through predicted ligand interactions (green). Red: mutated residues on the β-sheet or proximal regions showing low MR1 expression and/or inhibited MAIT cell activation; gray: ligand-interacting residues not mutated. **(B)** Structural interactions between MR1 protein and the ligand RL-6-Me-7-OH (green) (34). **(C)** Mutated mouse MR1 residues (84) are annotated on hMR1 crystal structure (34) to show the resulted functional impact on T cell activation through predicted interaction with MAIT cell TCR. Red: mutated residues on helical regions showing inhibited MAIT cell activation; gray: mutated helical residues not showing functional impact. **(D)** Surface MR1 residues that structurally interact with corresponding complementarity-determining regions (CDRs) are shown with the same color (34).

**Figure 3:**
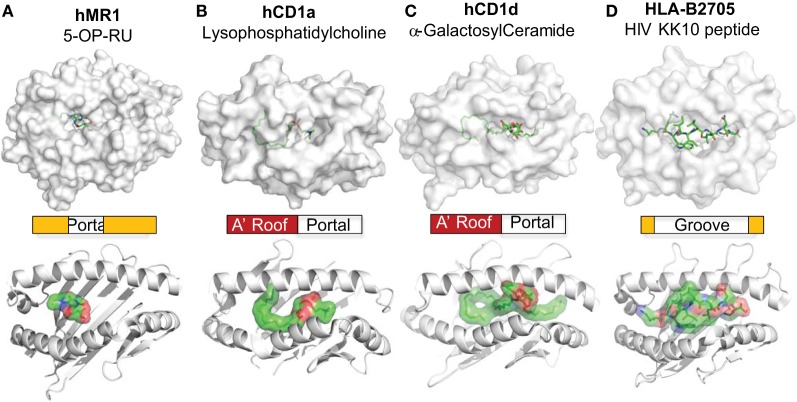
**MR1 and CD1 ligand-binding clefts**. The crystal structures of MR1 **(A)** (34), CD1a **(B)** (58), and CD1d **(C)** (134) molecules are shown with associated ligands (green) in comparison to the HLA-B2705 protein **(D)** (135). The positions of the portals for the MR1 and CD1 ligand-binding clefts are annotated. The shapes and relative sizes of the MR1 and CD1 ligand-binding clefts are also shown with ribbon view.

The MR1 ligands identified to date share one or two cyclic structures, which are embedded within the MR1 ligand-binding cleft through interactions with aromatic or basic residues of MR1 proteins (Figure 2). Different from the ligand-binding grooves of MHC class I or class II proteins, which are shallow and open to access by antigenic ligands, the MR1 ligand-binding cleft has a small open portal in the middle portion of the MR1 protein (Figure 2). The MR1 ligand is located centrally within the MR1 ligand-binding cleft in a very small region, with the cyclic structure toward the base of the β-sheet and the ribityl side chain positioned upward, as shown in MR1 structure with ligand RL-6-Me-7-OH (Figures 2A,B). Interestingly, similar to ligand binding in CD1 proteins (19, 91), antigen binding to MR1 proteins exhibits very limited solvent accessibility and is largely buried within the MR1 protein. The interaction between RL-6-Me-7-OH and MR1 protein is dominated by hydrophobic interactions, with Tyr 7, Tyr 62, Trp 69, and Trp 156 forming an “aromatic cradle” that sequesters the ligand (Figures 2A,B) (31, 34). The physical interaction between the MR1 ligand-binding cleft and the ligand is consistent with the functional result in a mutagenesis study that shows the mutation of Tyr 7, Arg 9, and Arg 94 reduces MR1 expression and MAIT cell activation (Figure 2A) (84). By taking a similar mode of interaction with the MR1 protein, both bicyclic and monocyclic moieties of MR1 ligands occupy a small area of the MR1 ligand-binding cleft (Figures 2 and 3A) (36), although RL-6-Me-7-OH occupies a large region of the cavity, making numerous contacts within the cleft to correctly orient for T cell recognition in the absence of Schiff base bond formation (34).

## Hydrophobic Anchor and Right-Sided Portal for CD1

CD1-lipid binding also shows a very different mechanism from the peptide antigen loading in classical MHC proteins. Lipid metabolites usually consist of aliphatic hydrocarbon chains present in the alkyl and polyketide tails with repeating methylene unites and hydrophilic head group (101, 136, 137). A lipid metabolite usually contacts with the non-polar residues on the inner surface of the CD1 ligand-binding cavity through non-specific hydrophobic interactions (138–140). With major aliphatic chains buried in the CD1 proteins, head groups of lipid antigens protrude out from the small portals to the right side of the CD1 ligand-binding cleft (Figure 3). Unlike the importance of the position of anchor residues for determining the orientation of peptide antigen binding to MHC molecules (141), the hydrophobic inner surfaces in CD1 proteins interact with proximal, center, and distal ends of the aliphatic chains of lipids biochemically independently of the position of the methylene unites. Although the binding of lipid ligands with CD1 proteins uses a more promiscuous, position-independent mode, the size of the clefts and individual pockets within the CD1 proteins places an upper limit on lipid antigen binding, as previously reviewed (142). However, this promiscuous interacting mode allows CD1 proteins to bind to a very broad spectrum of cellular lipids, leaving less restriction on lipid metabolite binding than on peptide binding to MHC molecules.

## Dual Presentation of Self and Non-Self Ligands

Whereas most models of T-cell recognition emphasize TCR discrimination of self and foreign antigens, the innate immune system recognizes both self and foreign ligands. Similar to PRRs, which have evolved to bind pathogen-derived and stress-induced specific molecular patterns (94, 95), MHC class I-like molecules also bind both self and non-self ligands (Table 1). For example, the CD1a protein binds both mycobacterial DDM (24) and endogenous lysophosphatidylcholine (LPC) (58). CD1b autoreactive T cells have been shown to recognize phosphatidylglycerol (PG) lipids derived from mammalian cells, *Salmonella, Staphylococcus*, and other bacteria (61). Alternatively speaking, CD1b did not discriminate the structural differences that distinguish mammalian PG from bacterial PG or distinguish *Salmonella* PG from other bacterial PG, supporting the dual recognition of self and bacterial lipids by CD1b-restricted autoreactive T cells. The CD1c- and CD1d-mediated presentation of both self and non-self lipids has also been observed (Table 1) (51, 67, 143). Moreover, Qa1 protein, a mouse homolog of human HLA-E protein, binds signal peptides derived from self class I molecules as NK sensors for viral infection as well as GroEL peptides from *Salmonella typhimurium* or mycobacterial peptides to stimulate a cytotoxic T-lymphocyte response against infection (Table 1) (144, 145). Interestingly, these GroEL-specific cytotoxic T-lymphocytes cross-react with the stress-induced HSP60 peptide as a potential danger signal (144, 145). Moreover, mouse MAIT cells have been demonstrated to respond to both endogenous and exogenous antigens (84, 100, 146–148). Human MAIT cells are reactive to bacterial antigens in bacterial infection, which likely also induce endogenous antigens, similar to the activation of CD1d-restricted NKT cells (51). It appears very common for innate-like T cells to be reactive to both self and foreign antigens, supporting an innate-like feature of immune response.

## Semi-Invariant TCR Beyond the Public TCR

TCRs of conventional T cells are expressed through rearrangement of the V, D, J, and C gene fragments of the β chain and the V, J, and C gene fragments of the α chain upon antigen stimulation. TCR gene rearrangement also accompanies T cell clonal expansion to proliferate a cluster of new T cells that express a rearranged TCR with specificity to bacterial priming antigens. While undergoing TCR rearrangement and clonal expansion in the priming stage, the primary response of conventional T cells is delayed, as in *M. tuberculosis* infection (Figure 1). However, public TCRs of conventional T cells can occur in small populations of donors sharing the same MHC allele in an infection of the same pathogen that expresses an immunodominant antigen (149). In this case, the same microbe must infect multiple individuals and prime T cell responses with the same antigens. These individuals should share similar HLA gene sequences to be able to present the same set of peptides. The generation of a public TCR in this setting is a highly coincident scenario and a rare event, as HLA alleles are highly variable in general human populations. Even though the public TCR is expressed, it yet exists in a very small human population. In contrast, the V and J rearrangements that define the MAIT and NKT TCRα chains are highly conserved in humans and mice. The invariant rearrangement of V and J gene fragments possibly occurs upon the stimulation of structurally conserved metabolites available prior to the infection of microbial pathogens. These semi-invariant TCRs are much more “popular” during the development and common in human populations than are the public TCRs (27, 150, 151). In addition to the public TCRs from conventional T cells, MAIT, NKT, GEM, and LDN5-like T cells express semi-invariant TCRs (Table 1). Unlike the fortuitously rearranged conventional TCRs, MAIT TCR is assembled by a canonical TRAV1-2-TRAJ33 (hVα7.2-Jα33 and mVα19-Jα33) chain paired with limitedly variable β chains TRBV6 and TRBV20 (114). The iNKT cell TCR is assembled by the TRAV10-TRAJ18 (hVα24-Jα18; mVα14-Jα18) chain mainly paired with TRBV25. Together with other invariant or biased TCR rearrangement (Table 1), MAIT and iNKT cells offer semi-invariant T cell receptors for the recognition of antigen-presenting molecules and antigens.

## Limited Interference of Metabolite Antigens

**Figure 4:**
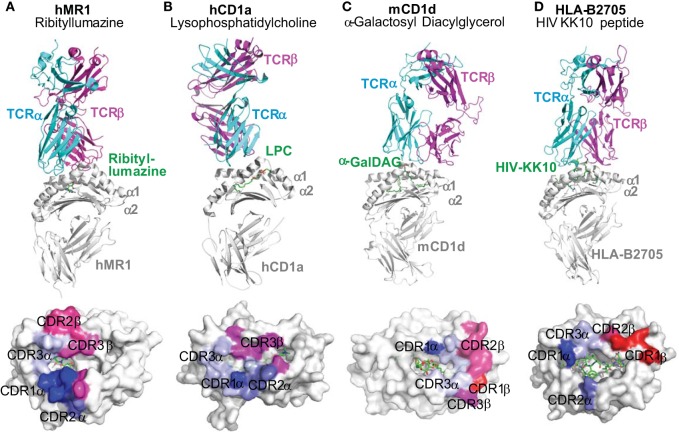
**Docking mode of T cell receptors to MR1, CD1, and HLA-B2705 proteins**. Tri-molecular interaction among an antigen-presenting molecule, an antigen, and a TCR in the activation of unconventional T cells is represented with the tri-molecular complexes of **(A)** human MR1-ribityllumazine–mucosal-associated invariant T cell TCR (34), **(B)** human CD1a–lysophosphatidylcholine–TCR of BK6 T cell (58), **(C)** mouse CD1d–α-galactosyl diacylglycerol (α-GalDAG)–iNKT TCR (134), and **(D)** HLA-B2705-KK10 peptide from human immunodeficiency virus Gag protein-TCR (135). The color-coated residues annotate the regions on the surface of antigen-presenting molecules to interact with CDR regions of TCRs (bluish colors for TCRα interactions and reddish colors for TCRβ interactions). TCRs from unconventional T cells more dominantly contact the surfaces of MR1 and CD1 proteins than the metabolite antigens. However, TCRs from conventional T cells recognize both HLA proteins and peptide antigens.

The docking mode of classical MHC molecules and TCRs utilizes various degrees of diagonal orientation through recognition of both peptide antigens and MHC proteins, as defined by the disulfide bond between TCRα and β chains with respect to the α1 and α2 helixes (19, 152, 153). In the MHC–peptide–TCR tri-molecular interaction for the activation of conventional T cells, multiple complementarity-determining regions (CDRs) of TCR usually come into contact with residues of antigenic peptides (19, 153). However, most contacting sites from the TCRs of MAIT cells and lipid-specific T cells interact with the surface of MR1 or CD1 proteins rather than the antigens (Figures 2 and 4). Thus, in a very different manner, the TCRs of lipid-specific T cells use an asymmetric mode with little interference from associated lipid metabolites to dock on the surface of the CD1-lipid antigen complex as reviewed (91). Briefly, the lipid metabolites associated with CD1 proteins are either amphipathic or purely hydrophobic. The amphipathic lipids, such as phospholipids and glycosphingolipids, typically use the hydrophobic aliphatic chain to crawl into the ligand-binding cleft of CD1 proteins, with the hydrophilic head group protruding outside of the portal of the ligand-binding cleft for TCR interactions (Figures 3 and 4). The non-amphipathic lipid metabolites, such as wax ester and squalene, are likely fully embedded within the ligand-binding cleft of CD1a protein from direct interactions with TCRs (59). Interestingly, upon lipid association with CD1 proteins, the portals of the ligand-binding cleft usually open to the right side and form a laterally asymmetric displaying platform. On a large portion of the CD1 protein surface, an “A’ roof” forms to the left side to cover the A’ pocket of CD1 proteins (91). Therefore, the TCRs of lipid-specific T cells are able to utilize an interacting mode called “limited interference” to contact CD1 surfaces without an exposed lipid motif (Figure 4B). For example, the BK6 TCR binds on the left side of the CD1a platform and exclusively contacts the “A’ roof” of the CD1a protein, without contact with the associated LPC or fatty acids within the CD1a ligand-binding cleft (Figure 4B) (58). Alternatively, TCR interacts with the right side of the CD1 antigen-displaying platform TCR recognition of CD1d–α-galacosyldiacylglycerol (Figure 4C) (134) or CD1d–α-galacosylceramide (154). Both manners of interaction provide the major portion of TCR contacting surface with “limited interference” from the embedded lipid antigen underneath (91) (Figure 3).

Similarly, the MAIT cell TCR mainly resides on the MR1 protein surface, although the direct interaction of MAIT TCR with the antigen appears critical. Human MR1/RL-6-Me-7-OH/MAIT TCR complex shows a hydrogen bond interaction between the Y95 residue at the TCR CDR3α region and the ribityl side chain of the antigen RL-6-Me-7-OH (Figure 2D and Figure 4A). The other stimulatory MAIT cell antigens with a ribityl side chain, such as 5-OP-RU and 5-OE-RU, also form a hydrogen bond with the TCR CDR3α region (34, 36, 155). The tri-molecular interaction has been further recapitulated with two additional xenoreactive human MR1/antigen/bovine MAIT TCR complexes (155, 156). Both ternary structures and functional studies suggest that the ribityl side chain is critical for TCR recognition and T cell activation (Figure 2D), as the ribityl side chain is unavailable to interact with MAIT cell TCR, 6-FP and Ac-6-FP are unable to directly contact TCRs and activate MAIT cells (37). It also appears that a Schiff base bond formation strengthens the binding of ribitylaminouracil (5-OE-RU and 5-OP-RU) to the MR1 protein and the tri-molecular interaction in TCR-5-OP-RU-MR1 and TCR-5-OE-RU-MR1 complexes, supporting a strong potency for MAIT cell activation (37).

Compared to the interaction between conventional TCR and peptidic antigens, TCR interaction with mostly embedded metabolite antigens is limited in terms of the number of contacting sites between TCR and antigens (Figure 4). Therefore, a “limited interference model” can be proposed to describe the degree of TCR–antigen interaction required for the activation of innate-like T cells. This model will not understate the importance of the interactions between TCR and some antigenic motifs, which are likely determinative for the activation of MAIT and iNKT cells by some antigens, such as the ribityl side chain for MAIT cell TCRs (37) and the galactose moiety for iNKT cell TCR (154). Functionally, a “limited interference model” better explains the quick activation kinetics in innate-like T cells, which are less dependent on the priming with structurally identical antigenic metabolites.

## Low Antigen Discrimination in Tetramer Detection

It is known that the tetramers formulated with classical MHC proteins and bacterial peptide antigens usually detect antigen-specific conventional T cells upon the infection of the corresponding bacterial pathogens (3). In contrast to this highly specific reactivity of tetramer staining using classical MHC proteins and bacterial peptides, the tetramers of MHC class I-like proteins for detecting innate-like T cells are highly conserved and appear to act in an antigen non-discriminative manner. Conservation of the semi-invariant TCR of innate-like T cells is reflected by the positive detection of MAIT or iNKT cells in uninfected human individuals using MR1 or CD1d tetramers with bacterial metabolite antigens (44, 45). Recently, hMR1-rRL-6-CH2OH (32), MR1-5-OPRU, and MR1-5-OE-RU tetramers (36) were used to efficiently detect human MAIT cells in peripheral blood mononuclear cells (PBMCs). The detected MAIT cells were considered to cover most MAIT cells in humans or mice with or without exposure to antigen-producing bacterial pathogens (33, 157, 158). It has also been claimed that MR1-5-OP-RU and MR1-5-OE-RU tetramers stain all human MAIT cells in peripheral blood, possibly because of the high affinity of these pyrimidine metabolites to interact with both MR1 protein and MAIT TCRs (36). Similarly, CD1d–α-GalCer tetramer also detects iNKT cells in healthy mice from “naïve” T cell populations (82, 159, 160). The tetramer recognition of innate-like T cells in both healthy donors and tuberculosis patients raises an interesting question regarding whether the tetramer-positive T cells in healthy and infected individuals function differently. Since the stimulatory antigens for eliciting MAIT cell and NKT cell responses can be derived from different bacterial species (32, 33, 157), the ability of mycobacterial metabolites to induce protective antimycobacterial immune responses could feasibly be compared with that of other bacterial metabolites.

## Xenoreactivity Beyond Alloreactivity

Cross-species activation confers an interesting innate-like manner of reactivity that is unlikely to exist in the adaptive immune system. In addition to broad alloreactivity in human populations, MHC class I-like molecules also generate very interesting cross-species reactivity in mammalian systems, as called xenoreactivity. Xenoreactivity is usually mediated by innate receptors such as NK cell receptors (161). These germline-encoded receptors are able to recognize conserved molecular motifs on targeted cells from different species; for example, human NK cells adhere to and lyse porcine cells (161). However, the activation of conventional T cells is determined by the recognition of both highly variable MHC molecules and heterogeneous peptide antigens (Figures 3D and 4D), which are unlikely to activate conventional T cells of a different species. By taking advantage of promiscuous and conserved tri-molecular interactions, MR1- and CD1-restricted T cells exhibit the interesting ability of cross-species activation. The xenoreactivity of T cells supports conserved structures and functions for antigen presentation (100). Indeed, the MR1 protein confers a high percentage of sequence homology among mammalian species, which is higher than 80% for rodents, bovines, and humans for the α1 and α2 domains of MR1 (100). Mouse MR1 is able to activate both mouse and human MAIT cells. Human MR1 with a single residue mutation to the corresponding residue in mouse MR1 (L151Q) is able to activate human MAIT cells (100). Moreover, the iNKTs in mice recognize the lipid antigens presented by human CD1d proteins. This is also true *vice versa* (162, 163), although CD1d sequences are not as homologous as MR1 proteins between mice and humans. Structural analyses of NKT TCRs responding to mouse and human CD1d–α-GalCer complex reveal that a contiguous CDR3β sequence is conserved between humans and mice to provide structural plasticity to accommodate a variety of glycolipid antigens presented by CD1d (164). Similarly, in comparison to conventional peptide-presented T cells, peptides presented by human HLA-E bind to the mouse homolog Qa-1b molecule (165).

## Proliferation and Exhaustion

The available number of effector cells and the effectiveness of each effector are important criteria for estimating the final efficiency of immune cells in antimycobacterial immune defense. Conventional T cells specifically respond to bacterial peptide antigens and undergo clonal expansion to achieve multiple sets of functional capacity in immune defense, which include: (i) effector functions for cytokine production or cytolysis; (ii) memory functions for long-term antimicrobial immune responses; (iii) ability to amplify their cell number from an undetectable or absent state to high frequency; and (iv) ability to migrate from antigen-priming tissues to lesion tissues, for example, from lymph nodes to mycobacterial-infected alveolar tissues. A clonal expansion is usually required for conventional T cells to achieve a sufficient number of effector T cells upon antigen stimulation. In the case of aerosol infection with *M. tuberculosis*, mouse CD8^+^ T cells positive for the tetramer H-2Kb-TB10.3/10.4 will proliferate from undetectable to 1 million cells/lung, peak around 4 weeks after primary infection, and have an 8- to 12-fold increase in secondary challenge with the aerosol infection in comparison to the unchallenged (166). Thus, the clonal expansion of conventional T cells is vast upon antigen stimulation.

However, the cell frequency change of MAIT cells in *M. tuberculosis*-infected individuals is distinct from that of conventional CD8^+^ T cells. MAIT cells dramatically decrease in peripheral blood and are likely increased in lung tissues, as demonstrated in active *M. tuberculosis*-infected patients, suggesting the migration of activated MAIT cells to infected tissues (12). In Vα19iCα^–/–^MR1^+/+^ mice, MAIT cells accumulate three-fold more in BALF in comparison to that found in Vα19iCα^–/–^MR1^–/–^ mice, as detected with MR1-rRL-6-Me-7-OH and MR1-5-OP-RU tetramers (33). With a high basal frequency of precursor MAIT cells prior to primary infection, the clonal expansion of MAIT cells upon mycobacterial infection appears much weaker than the clonal expansion of conventional T cells. Without undergoing potent clonal expansion, MAIT cells are ready to exhibit effector functions upon activation, although further expansion, migration, or reduction of precursor cells can occur.

Whether the lower frequency of MAIT cells in tuberculosis patients is attributable to MAIT cell exhaustion is an interesting question. When MAIT cells are analyzed in patients with pulmonary tuberculosis, tuberculous pleurisy, and tuberculous peritonitis by flow cytometry, a dramatically reduced MAIT cell number is usually observed. On the one hand, patients with active tuberculosis have a significantly higher production of cytokines IFN-γ and TNF-α from MAIT cells in responding to *ex vivo* BCG stimulation but not to *E. coli* stimulation, as compared to healthy control subjects (167). On the other hand, it is interesting that MAIT cells in patients with active tuberculosis exhibit an elevated expression of programed cell death protein-1 (PD-1), and the blockade of PD-1 signaling results in a significantly higher frequency of BCG-stimulated IFN-γ production from MAIT cells (167, 168). Whether the expression of PD-1 protein in MAIT cells indicates an exhaustion phenotype is controversial in regard to tuberculosis, particularly considering that PD-1^–/–^ mice are sensitive to *M. tuberculosis* infection (169). However, the decreased frequency of MAIT cells may also be due to a decreased expression of CD161 in the existing MAIT cells rather than to an actual reduction in the MAIT cell number. Exhaustion or apoptosis in NKT cells has also been observed. The poor response to α-GalCer in *M. tuberculosis*-infected patients has been found to be due to increased NKT cell apoptosis, reduced CD1d expression, and a defect in NKT cells (170). Similarly, *M. tuberculosis* infection is associated with an elevated expression of the inhibitory PD-1 receptor on NKT cells, and the blockade of PD-1 signaling has been shown to enhance the response to α-GalCer.

## Innate-Like T Cells in Bacterial Killing and Cytokine Production

**Figure 5:**
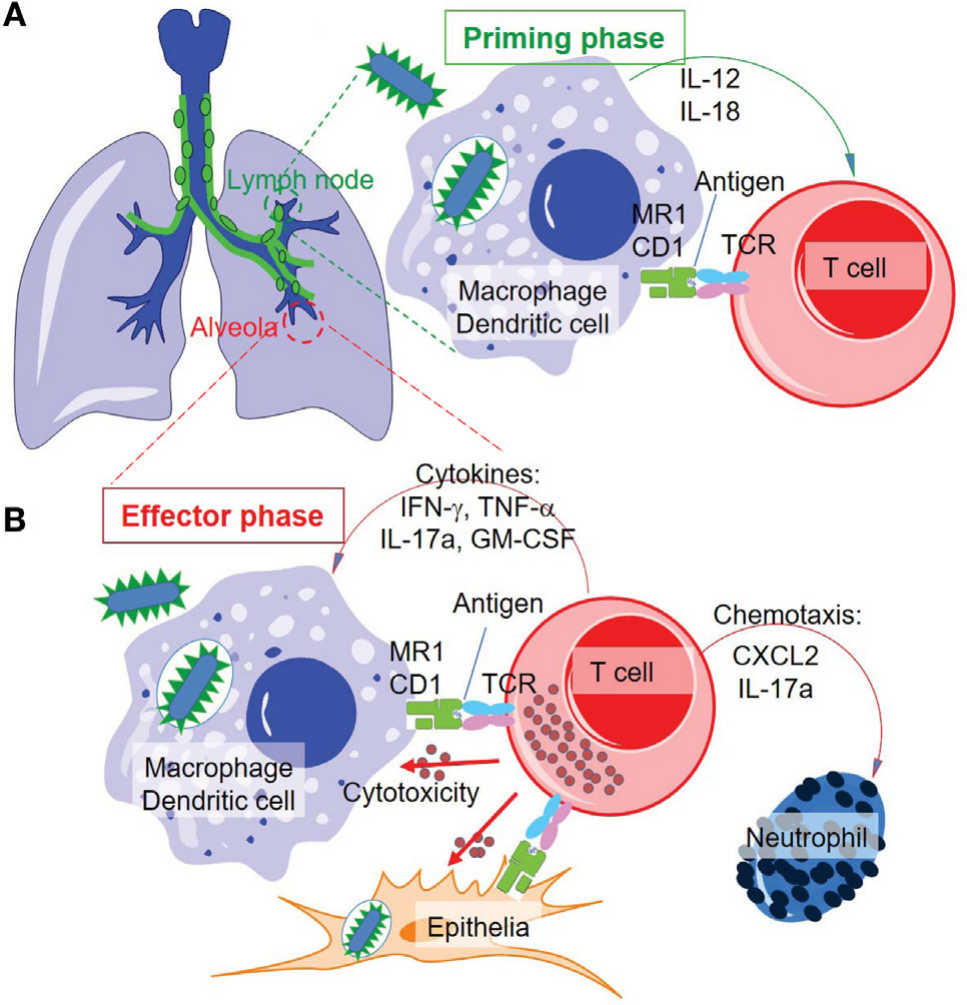
**Proposed priming and effector phases of innate-like T cell responses in tuberculosis**. **(A)** The priming phase may occur in the lymph nodes of lung tissues. Naïve or precursor innate-like T cells are activated through interaction with MR1 and CD1 proteins and/or stimulation of cytokines. **(B)** Activated innate-like T cells migrate to infected tissues, such as alveolar regions, to perform cytotoxic function and secrete cytokines and chemokines in anti-*M. tuberculosis* effector response.

Effector functions of MAIT cells contributing to protectivity against mycobacterial infections have been recently demonstrated upon MAIT cell activation (Figures 5A,B). MAIT cells isolated from the thymus and peripheral blood have shown the ability to kill mycobacterial-infected antigen-presenting cells *in vitro*. Pathogen-unexposed MAIT cells respond to lung epithelial cells infected with *M. tuberculosis* and produce tumor necrosis factor-α (Figure 5) (12, 86). As a dominant effector cytokine secreted by MAIT cells in most *in vitro* assays, TNF-α is an inflammatory cytokine and plays a critical protective role against mycobacterial infection, at least in the early stage of infection. The protectivity of TNF-α is supported by the fact that chemical blockers of TNF-α used for treating rheumatoid arthritis have caused the reactivation of tuberculosis (171). However, TNF-α also has the potential to worsen the inflammatory pathology at a later stage as a “double-edge sword.” Thus, maintaining an advantageous balance between TNF-α-mediated protective functions and pathogenic outcomes is critical in *M. tuberculosis* infections (172, 173). MAIT cells also express other pro-inflammatory cytokines, including IFN-γ and IL-17 (Figure 5B) (20, 30, 83). When pathogen-unexposed mouse MAIT cells are cocultured with BCG-infected macrophages, MAIT cells are quickly activated, as reflected by the production of large amounts of IFN-γ and IL-17a. Cytokines IFN-γ and IL-17a contribute to controlling BCG growth in macrophages, as supported by the effect of anti-IFN-γ and anti-IL-17a antibodies on impairing macrophage immunity (Figure 5B) (80). IFN-γ is also a critical antimycobacterial cytokine produced by conventional CD8^+^ T and CD4^+^ T cells (2) that has demonstrated a protective function in most stages of mycobacterial infections. While it is produced by MAIT cells, IL-17a is able to inhibit BCG growth in infected macrophages (83). However, the long-term impact of IL-17 in *M. tuberculosis* infections remains unclear (2). Moreover, cytotoxic reactivity allows MAIT cells to lyse *Shigella flexneri*-infected epithelial cell line HeLa cells (174) and *M. semegmatis*-infected lung epithelial A549 cells (175). MAIT cells upregulate the expression of perforin, granzyme B, and surface CD107a (LAMP1) upon the stimulation with anti-CD3 and CD28-coated beads, supporting their cytolytic function (Figure 5B) (174). Similar to NK and NKT cells, a high percentage of MAIT cells express the CD161 molecule, which is believed to modulate the cytokine response, such as IFN-γ, TNF-α, and IL-17 cytokine secretion (174, 176). But CD161 may not regulate the cytotoxic activity of MAIT cells (174). This is unlike the inhibitory effect of the CD161 molecule previously shown on NK and CD8^+^ T cells (177, 178). Overall, MAIT cells have a mixed Th1/Th17-associated pattern of cytokine production, including TNF-α, IFN-γ, and IL-17 at a striking level similar to conventional memory T cells, to inhibit mycobacterial growth in infected macrophages (30, 83). For *in vivo* mycobacterial infections in MR1 knockout or MAIT cell over-expressing mice, MAIT cells have shown the capacity to decrease bacterial loads (20, 33, 83, 158, 174), upregulate the Th1-, Th2-, or Th17-like cytokines, and enhance their cytotoxicity and frequency.

Similarly, overall observations show that NKT cells have a decreased frequency in peripheral blood and a weaker response in human *M. tuberculosis* infection (170) and HIV infection (179). When cocultured with splenocytes isolated from uninfected mice, *M. tuberculosis*-infected macrophages suppress bacterial replication in macrophages dependent on the function of iNKT cells (81), which is similar to the suppression of BCG replication in macrophages by MAIT cells (80). Cytotoxicity, IFN-γ, and GM-CSF production have been shown upon activation of iNKT cells (81, 180). The adoptive transfer of naïve splenic iNKT cells from uninfected mice significantly reduces the bacterial burden in the lungs of infected mice (81). This effector function and protectivity does not require *in vitro* or *in vivo* stimulation of iNKT cells with a strong agonist of α-GalCer as shown previously (89). Thus, in therapeutic development, it would be interesting to further understand whether endogenous or mycobacterial lipid antigens are critical for CD1d-mediated iNKT cell effector function. In addition to the direct effect of bacterial inhibition and killing, the stimulation of HLA-E-restricted T cells and CD1d-restricted T cells is able to enhance the secretion of Th2 cytokines (IL-4, -5, -10, and -13), regulating B-cell activation and antibody production in antimycobacterial immune defense (181–183).

However, the role of lipid-specific T cells restricted by group I CD1 proteins in antimycobacterial effector responses is less understood. Several early studies showed that the frequency of group I CD1-restricted T cells increases in *M. tuberculosis*-infected individuals, suggesting the activation and expansion of *M. tuberculosis*-specific CD1-restricted T cells (23, 64, 184). The overall effector responses of lipid-specific T cells restricted by group I CD1 proteins are similar to those of iNKT and MAIT cells and are characterized by the production of cytokines IFN-γ and TNF-α upon stimulation with mycobacterial lipid antigens. Since mice do not express group I CD1 proteins, CD1a, CD1b, or CD1c, a study using a guinea pig model expressing human CD1b and CD1c homologs (185, 186) showed that immunization with mycobacterial lipids elicits the antigen-specific proliferation and cytotoxicity of group I CD1-restricted T cells (187) and minimizes the lung tissue damage induced by *M. tuberculosis* challenge (188). However, group I CD1 protein responses to *M. tuberculosis* infection exhibit relatively delayed kinetics in responding to mycobacterial lipid-pulsed mouse DCs, as shown using a humanized mouse model expressing human CD1a, CD1b, and CD1c proteins (90). This response peaks 7 days after immunization in unexposed mice, but it is difficult to compare this kinetics with those of conventional T cells because of the different immunization approaches used in various experiments. For example, the memory responses of DO11.10 T cells in mouse draining lymph nodes to the OVA peptide expressed in infected *Salmonella typhimurium* take about 5 days (189). Thus, whether group I CD1-restricted T cells utilize fast or slow kinetics in primary immune response remains unclear. Further, tetramer staining of group I CD1-restricted T cells seems to be a promising approach to determine the frequency and antigen specificity of T cells. Current reports of tetramer staining in infected and healthy human blood samples mostly show a minimal frequency of CD1a and CD1b tetramer-positive T cells (67, 190–192). The underlying mechanism may be multi-dimensional, for example, more immunodominant or conserved antigens may exist to detect a higher frequency of group I CD1-restricted T cells, or an antigen re-stimulation may be needed to expand the number of group I CD1-restricted T cells. However, CD1c loaded with phosphomycoketide lacking a carbohydrate head group is able to stain a high percentage of polyclonal T cells in the blood samples of donors with latent *M. tuberculosis* infection (67), suggesting the potential to detect a high frequency of some group I CD1-restricted T cells in patients.

## Memory Phenotype

Memory T cells are T cells that can be quickly reactivated and become responsive to bacteria immediately after infections (193). The described relative quick kinetics for activated innate-like T cells in lung tissues is consistent with the memory phenotype of innate-like T cells. Although the kinetics of lung T cell activation can be impacted by the delayed or detrimental growth of *M. tuberculosis* in infection (194, 195), the memory phenotype of innate-like T cells can be still reflected by the expression of surface memory markers. It has been shown that these semi-invariant MAIT cells express memory markers (mainly CD45RA^low^CD45RO^high^) in humans without additional *in vitro* stimulation (150). Further detailed phenotyping of MAIT cells using a MAIT cell over-expressing mouse model, Vα19^+^Ca^−/−^MR1^+/+^, has indicated that a high percentage of MAIT cells are CD44^high^CD45RB^low^CD62L^low^CD25^high^ (196) upon TCR ligation with anti-CD3 and anti-CD28 antibodies. Recently, multiple reports further confirmed the expression of memory markers CD44^high^CD62L^low^ in mice (158) and the expression of CD45RA^−^CD45RO^+^CD95^high^CD62L^low^, an effector-memory phenotype, in humans (30, 197, 198). Understanding the acquisition of this memory phenotype over the lifetime of humans is critical for determining the immune therapeutic strategy upon mycobacterial infection. In adults, MAIT cells have an effector-memory phenotype (largely CD45RA^−^CD45RO^+^CCR7^low^CD62L^low^) (199). In cord blood, MAIT cells express the markers of naïve cells (CD45RA^+^CD45RO^−^) (200, 201). These cells are also CCR7^low^CD62L^low^, suggesting that CD45RO positive expression is obtained after birth, which is consistent with the stepwise MAIT cell development facilitated by the maturation of gut microbiota (200). These critical observations of the acquisition of an effector-memory phenotype during the stepwise development of MAIT cells support the notion that antigen stimulation in the early lifetime facilitates the formation of the memory phenotype. It is likely that the microbial cyclic small molecules, such as riboflavin precursor metabolites defined at later time (31, 36), facilitate the process of memory formation. Similarly, NKT cells from the pleural fluid mononuclear cells of *M. tuberculosis*-infected patients also express CD45RO^high^CD62L^low^CCR7^low^ that supports an effector memory phenotype for NKT cells (202).

The determination of whether microbial antigens are involved in the acquisition of the memory phenotype of MAIT cells helps in answering the question of whether MAIT cells are pre-activated by commonly distributed cross-species conserved antigens. This pre-activation is likely a major mechanism through which MAIT cells acquire an activated or memory phenotype and allows MAIT cells to be ready to respond to stimulation by different and unexposed pathogens. Similar or identical conserved antigens shared between gut microbiota and pathogens, such as riboflavin precursor metabolites, may be driving elements for the formation of memory or activated phenotypes of MAIT cells. Thus, the activated and memory phenotypes of MAIT cells in adults may have been shaped by the stimulation of microbial antigens during the early stage of individual development. The term “innate-like T cells” broadly defines the pre-activated memory phenotype of T cells and a quick activating kinetics upon encountering previously unexposed pathogens that potentially express metabolite antigens with the same or similar chemical structures to the “pre-activating” metabolites. The quick activation kinetics and memory phenotype of innate-like T cells suggest that the effector and regulatory functions of innate-like T cells may occur prior to the activation of the adaptive immune system (Figure 1).

## Tissue Tropism in Early Defense

In *M. tuberculosis* infection, a rapid and regional immune response is important for containing the bacteria and infected cells in the initial stage of infection. Upon aerosol infection with *M. tuberculosis*, the acquired cellular immune responses are slow to be induced and take effect within the lung (2). This lagged period allows the invading slow-growing *M. tuberculosis* to grow and initiate conventional T cell activation. The stimulation of conventional T cells against *M. tuberculosis* requires the interaction of antigen-presenting cells, bacteria, and T cells in lung tissue or draining lymph nodes. Failure in this battle in lung tissue will lead to bacterial outgrowth, host pathology, and bacterial dissemination to distal tissues. In a beneficial response, around 8–9 days post mycobacterial infection, dendritic cells and macrophages will sample *M. tuberculosis* and migrate to draining lymph nodes (Figure 5A), and naïve T cells will be primed to proliferate and become effector cells. The activated effector cells will reversely migrate to the lung tissue at around 18–20 days post infection as a consequence of inflammatory responses to kill infected phagocytes and produce cytokines (2). MAIT cells have been shown to accumulate in lung tissues but decrease in peripheral blood in infected humans, indicating a mechanism allowing MAIT cell migration to lung tissues (12). Although the *in vivo* kinetics of MAIT cell stimulation upon mycobacterial infection are not clear, an initial assay using BCG-infected mice suggests that MAIT cells are critical for protecting mice from the high bacterial burden at day 10 but not at day 30 following infection (80), which is consistent with the protection conferred by the innate-like responding kinetics. Further *in vivo* studies are needed to determine the protection and early activation kinetics of MAIT cell responses to mycobacterial infections. In parallel, the role of group I CD1-restricted T cells will likely be further understood using animal models expressing group I CD1 proteins and having similar pathologies to mycobacterial infection in humans. Thus, the gap between the dissemination of bacteria and the onset of conventional T cell responses (2, 203, 204) is ideally filled by the responses of innate-like T cells. It seems promising that innate-like T cells could contain *M. tuberculosis* in the early stage of infection and contribute to a reduced rate of active disease in humans.

## Therapeutic Value and Remaining Questions

Targeting innate-like T cell activation will provide novel therapeutics applicable to various human populations for controlling the early stage of mycobacterial infection, in a manner complementary to conventional T cell-based therapies. Multiple lines of evidence support the unique therapeutic values of innate-like T cells in tuberculosis. First, an effective early T cell response likely leads to a low lifetime risk of developing active tuberculosis (3, 10). Second, innate-like T cell populations, especially MAIT and iNKT cells are ideal candidates to offer a protective effect in the early stage of mycobacterial infections (83, 180, 205). Third, previous application of α-GalCer in the early stage of *M. tuberculosis* infection was shown to protect mycobacterial-infected mice against a high bacterial burden and severe pathology (89). This protective effect is encouraging for further investigations of other vaccine strategies based on innate-like T cells. For MAIT cells, the mouse study supports the protection of MAIT cells in *M. abscessus*-infected mice (20) and a newly published study shows the upregulation of cytotoxic MAIT cells in BCG-vaccinated macaque (206). In humans, high frequency of MAIT cells in latent infections of tuberculosis or healthy donors and low frequency in active tuberculosis support the association of high MAIT cell frequency with healthy conditions (12). Fourth, current vaccine candidates in the clinical trial pipeline mostly target mycobacterial secretory proteins and induce dominant responses by conventional CD4^+^ and CD8^+^ T cells (14). To overcome the variation in generating conventional T cell responses in large human populations, as demonstrated in a recent clinical trial in South Africa (207–209), innate-like T cells with donor-unrestricted features are expected to be applicable in most or all human populations if showing effectiveness in small groups of donors. Moreover, in comparison to these gene-based or microorganism-based vaccination strategies (14), the application of small metabolite antigens for activating innate-like T cells is expected to be safer, as naturally existing small molecules are not genetic materials that potentially induce inheritable complications. Metabolite-activated innate-like T cell are abundant T cell populations and the protein-based vaccines in clinical trial pipeline (16) will miss these abundant targets, demanding the need of new strategies for antimycobacterial vaccine design (89).

However, it is critical to further understand the *in vivo* responding kinetics and protectivity of innate-like T cells or donor-unrestricted T cells in controlling *M. tuberculosis* infections, especially chronic infections. Approaches are needed to improve the efficiency of the antimycobacterial T cell responses mediated by innate-like T cells, for example, reducing the frequency of exhausted MAIT cells in active tuberculosis. Moreover, it is important to understand whether *M. tuberculosis* expresses unique small molecule antigens for inducing different MAIT and iNKT cell responses, similar to those induced by other bacterial antigens. What other conserved or unique antigens are able to induce the protective function of these MR1- and CD1-restricted T cells? Answering these questions will facilitate targeting candidate metabolite antigens and innate-like T cells for developing novel vaccination and therapeutic strategies against tuberculosis.

## Author Contribution

The author confirms being the sole contributor who analyzed the literature and wrote the manuscript.

## Conflict of Interest Statement

The author declares that the research was conducted in the absence of any commercial or financial relationships that could be construed as a potential conflict of interest. The reviewer AK and handling Editor declared their shared affiliation, and the handling Editor states that the process nevertheless met the standards of a fair and objective review.
